# Monitoring health determinants with an equity focus: a key role in addressing social determinants, universal health coverage, and advancing the 2030 sustainable development agenda

**DOI:** 10.3402/gha.v9.34247

**Published:** 2016-12-16

**Authors:** Nicole B. Valentine, Theadora Swift Koller, Ahmad Reza Hosseinpoor

**Affiliations:** 1 Department of Public Health, Environmental and Social Determinants of Health, WHO, Geneva, Email: valentinen@who.int; 2 Gender, Equity and Human Rights, WHO, Geneva, Email: kollert@who.int; 3 Department of Information, Evidence and Research, WHO, Geneva, Email: Hosseinpoora@who.int

In the 2030 Sustainable Development Agenda (SDA), population health is characterized as an intersectoral development challenge for all countries and for the international community as a whole (1). Health-related targets are therefore placed throughout the Sustainable Development Goals (SDGs) with the aim of highlighting the contribution of action across sectors to health and health justice, also referred to as ‘health equity’. Health indicators make explicit reference to reductions in morbidity, mortality, or burden of disease. Health-related indicators make explicit reference to improving the coverage of health services and reducing harmful physical exposures related to water, pollution, chemicals, violence, and climate change, to health states (e.g. malnutrition) and to health and health service events (e.g. coverage, births) (2, 3). In all, the SDA contains 23 health and ‘health-related’ targets of which 13 are in SDG goal 3 for health, with 35 indicators (26 of which are in this health goal) (2, 3).

The other 10 ‘health-related’ targets are spread across five other goals and in this way are intended to motivate other sectoral partners to take action to address health determinants. Yet aside from these, many other targets also have profound effects on health, and, in particular, on health equity. For example, there is an enormous body of evidence supporting the role of cash transfer policies in promoting health equity; yet the SDG social protection target and one of its proposed indicators, ‘Proportion of population covered by social protection floors/systems’ [SDG 1.3.1], is not listed as ‘health-related’ *per se* (4–6).

There is a clear need to base monitoring systems on a broader defined set of determinants that are important for health (7), while supporting efforts to have more health-related indicators included in the goals of other policy sectors. These are two related but separate streams of work. The former implies the need for a clearer position on *all* important determinants to hold multiple policy actors to account for their health impacts, even if partial or sophisticated measures, such as exposure rates or the attributable fraction of burden of disease, are not available.

This proposition also moves the focus from issue-specific health governance (e.g. tobacco, non-communicable diseases, climate change, and air pollution) to systemic approaches, like Health in All Policies (8), and to a focus on consequent implications for health systems’ monitoring, with particular emphasis on the health sector's public health monitoring function. The monitoring of health determinants, as characterized in this issue, can be thought of as a type of public health surveillance that focuses on upstream socioeconomic, environmental, and governance aspects determining population health and health equity. Public health surveillance is defined by the Centers for Disease Control and Prevention as ‘the ongoing, systematic collection, analysis, interpretation, and dissemination of data regarding a health-related event for use in public health action to reduce morbidity and mortality, and to improve health. Data disseminated by a public health surveillance system can be used for immediate public health action, program planning and evaluation, and formulating research hypotheses’ (see www.cdc.gov/mmwr/preview/mmwrhtml/rr5013a1.htm, and similar for WHO, see: www.who.int/topics/public_health_surveillance/en/).

There is uneven international guidance for national governments on health systems’ monitoring functions as they extend to health determinants. The World Health Organization (WHO) global monitoring documents make limited reference to monitoring of the more upstream determinants (9). Yet there has been no in-depth discussion of these systems combining these indicators (10). The latest WHO *2016 World Health Statistics* report represents a considerable step forward in advancing more systematic discussion of the health determinants. In previous WHO reports, health determinants were referred to as ‘risk factors’ or other ‘demographic and socioeconomic statistics’ (11–13). In order to promote health equity, it is important to have in place national monitoring systems that address health determinants (14). The evidence base shows that the social gradients in health pervade, even in countries where extensive national health systems are in place, implying an important role for other determinants of health (15).

The papers compiled for this *Special Issue* of *Global Health Action* address the theme of monitoring health determinants, and how this monitoring function can contribute to promoting action to address equity (‘justice’), human rights, and gender equality in health (16). The papers derive from work undertaken at WHO with a network of consultants between late 2013 and early 2015, prior to the finalization of the SDGs. At that time, the early think pieces on the post-2015 development agenda described the emergence of an intersectoral vision of health. Health sectors in countries would need to adapt their monitoring functions to embrace this intersectoral vision of health. While the vision for public health surveillance in the 21st century had been a topic of discussion for specific countries (e.g. USA) (17, 18), no global guidance existed. The work was seen as a way of addressing this gap to support implementation of the SDGs (19). The monitoring context which is discussed in these papers is largely at the national level. Studying the national level in light of the upcoming SDGs was considered important as the SDA effectively negotiated an international agreement on a common set of national development goals across policy sectors within and across countries. In the SDA discussions, the international health community, led by WHO and the World Bank, also singled out universal service and financial coverage of the population (‘Universal Health Coverage’ (UHC)) for emphasis by governments in the SDGs (20). As a result of these discussions, a special subtheme of the work was added to cover the implications of monitoring health determinants with respect to UHC (21). This subtheme is also reflected in the papers discussed below.

The papers in this *Special Issue* specifically focus on the scope of indicators needed in national monitoring systems if they are to address the determinants of health equity, and also how related analyses would be perceived by policy-makers. Before describing the contribution of each of the papers to this subject, a brief outline is given on the development of the indicators and the proposed analytical orientation of the monitoring framework for equity-oriented analysis of linkages between health and other sectors (termed the ‘EQuAL’ framework). The scope of policy areas was initially identified on the basis of literature reviews which covered evidence of causal pathways for health inequalities, a rapid review of data availability, and expert advice from workshops and key informants.

Following the philosopher Amartya Sen's conceptualization of both process (e.g. social justice) and capacities (e.g. material conditions) (22), as well as the General Comment No. 14 on *The Right to the Highest Attainable Standard of Health*, which emphasizes civil, political rights (e.g. participation), as well as economic, social, and cultural rights (e.g. access to food) (23), it was determined at the outset that monitoring should refer to a broad spectrum of conditions of daily life that promote health, including but beyond health care and material conditions. The final set of indicators covered 12 measurement domains aligned with typical national sectoral ministries and their policy mandates (and different SDGs): Income and poverty (SDG 1, 2), Knowledge and education (SDG 4), Housing and infrastructure (SDG 6, 7, 11), Travel (SDG 11), Community and infrastructure (SDG 9, 12), Social protection and employment (SDG 1, 8), Early child development (SDG 4), Gender norms (SDG 5), Participation (SDG 16), Registration (institutional constraints) (SDG 16), Accountability (institutional constraints) (SDG 10, 16), and Discrimination (SDG 5, 10). Given the abovementioned emphasis on UHC in the SDG agenda, it was determined that indicators should also expose barriers related to health services. Following a workshop held to discuss EQuAL, other criteria were introduced (16). This provided scope to include the so-called ‘policy’ indicators, which are also important for monitoring human rights and ensuring the accountability of policy-makers. This recognizes that policies can be changed within the policy-makers’ time of office (whereas outcomes and conditions may have longer lag-times) (16). Finally, the monitoring of health determinants was considered to be linked to, but separate from, monitoring inequalities in coverage and health outcomes (including through the enhanced availability of high-quality, timely and reliable data disaggregated by income, sex, age, race, ethnicity, migratory status, disability, geographic location and other characteristics relevant in national contexts (as called for by the associated SDG 17 target).

**Fig. 1:**
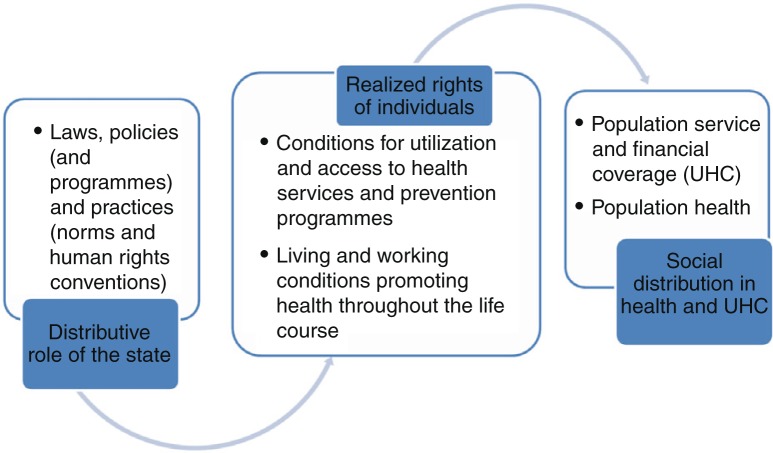
Important aspects of the scope of monitoring health determinants and its linkages with health and UHC inequality monitoring.

Figure 1 visualizes how all these concepts provided the broad parameters for the equity-oriented health determinants monitoring proposal on which the papers in the *Special Issue* are based.

The first paper in this *Special Issue* intentionally addresses the area of monitoring health inequalities in order to not only distinguish this area from monitoring determinants but also to show its complementarity and illustrate an approach to guidance and tools that may also be possible for monitoring health determinants. The paper by Hosseinpoor et al. (24) describes the extent of health inequalities monitoring, both globally and nationally, and the available tools for capacity building. A substantive volume of guidance and practical tools have been produced by WHO to assist countries with national monitoring of health inequalities. In order to advance action on health determinants, making health inequalities visible is a key step (25). Monitoring health inequalities has two functions. First, it makes the extent of health injustices visible. Second, where backed by evidence on the linkages between outcomes and determinants, inequality monitoring provides tools for visualizing the linkage between health and a subset of health determinants in a simple yet powerful way. For example, infant mortality stratified by maternal education principally describes social injustice; however, this can also be used as a tool to communicate the causal pathway related to lower health literacy. The approach to monitoring service coverage inequalities follows several of the principles developed for monitoring health outcomes (26).

Four countries were invited to test the EQuAL framework through the production of case studies that used largely qualitative techniques to assess the domains, indicators and analytical framing for feasibility, reliability, validity, and usefulness to policy-makers. The paper by Blas et al. (27) describes this process and the results obtained from four countries: Brazil, Bangladesh, Vietnam, and South Africa. The assessment describes how the country research teams found the data, developed the indicators, and discussed the domains using policy narratives with policy-makers from within the health sector and beyond. In general, there was cross-case support for the domains, but the case study authors indicated that the link between some indicators and public health goals were more difficult for target audiences to understand, in particular for the EQuAL domains oriented to civil and political rights (e.g. Participation and Accountability). The case studies noted that many issues covered by the domains were not institutionalized in data collection, analysis, or discussion in national systems. The authors propose that capacity building would be necessary in the countries studied in order to institutionalize equity-oriented monitoring of health determinants (27).

Following production of these four case studies, the individual country research teams were invited to develop a quantitative analysis to test the usefulness of the EQuAL framework as a tool for analysis. Each research group chose a different analytical emphasis for their study. The broad emphases discussed as part of the EQuAL project were 1) the presentation of ‘dashboards’ of indicators; 2) stratification of determinants, intersecting areas of stratification (e.g. transport/travel by education by sex), and the calculation of composite indicators for certain domains (for the housing and infrastructure domain, a composite for energy, water, sanitation, and waste removal was proposed); 3) multi-level analyses in evaluating the relative effects of different determinants; 4) the role of determinants of health and health inequalities versus determinants acting as health service ‘barriers’; and 5) the association of determinants with different types of health outcomes (e.g. non-communicable, reproductive/maternal, and communicable) and health services (e.g. preventive, treatment). Using regression analyses, the research teams were asked to compare the different determinants and the implications for intersectoral policy advice.

For Brazil, Rasella et al. (28) placed an emphasis on dashboards and intersecting inequalities in descriptive analyses. They developed indicator tables and mapped displays to describe inequalities in health determinants (stratified according to income quintiles, urbanization, race, and geographical region). In discussing results with policy-makers and comparing the indicator framework with existing monitoring efforts, the researchers concluded that monitoring and evaluation practices could be improved using the set of indicators assessed in their study, especially from a health equity perspective.

The Bangladesh research group investigated the area of neonatal, infant, and under-five child mortality and emphasized the use of multi-level analysis in their methodology. They used multilevel logistical regression modeling that adjusted determinants by level (children, nested in households, with mothers, and in sampling community clusters). The data allowed them to compare three time periods in Bangladesh. The regression odds ratio for childhood mortality was highest among children of young mothers, of parents with lower education, of mothers who lacked the power to take decisions about health-related matters of their children, and of poorer households. Here, poverty was a multidimensional measure, which could explain why, at the individual level, housing was not considered a statistically significant variable. The employment status of the mother was not significant either. At the community level, children born in rural communities or living in areas with roads that were not accessible in all seasons, had a higher risk of death.

In the study from Vietnam, Van Minh et al. (29) focused on developing indicators of intersecting disadvantage and composite indicators and also conducted regression analyses comparing determinants of health outcomes and barriers to service coverage. The composite barrier indicator performed well. Their results stressed the role of key determinants, namely, education and access to basic amenities in creating inequitable barriers to health services, thereby jeopardizing progress toward UHC goals. In particular, barriers for ethnic minority communities living in rural areas were prominent.

Finally, for the South Africa case, Ataguba et al. (30) described association between the determinants with a single more general measure, self-reported health. The advantage of this measure is that it reflects the health of the broader adult population, compared with the other country papers, which focused on particular population groups, and on reproductive and child health. Decomposition analysis was used to measure the relative contribution of various determinants to adult health inequality. The authors ranked social protection and employment, followed by knowledge and education, and then housing and infrastructure, as having the most important contributions to self-assessed health. They argued that their results provide a motivation for promoting actionable policies across the different responsible sectors. For example, the Ministry of Labour could improve implementation of active labor policies, the Ministry of Housing could improve the provision of social housing, and municipal governments could improve access to basic amenities. Although all the above national studies used cross-sectional designs, their results provide a promising start to understanding the scope of possible analyses relating determinants (or variables) to a range of different health outcomes, from across the life course, and to a range of important service coverage variables.

A paper by Valentine and Bonsel (31) explains different models for describing associations between determinants and health and coverage outcomes, including area-level inequalities. While their paper is applied to multi-country data, the modeling approaches are applicable to subnational area-level analyses. The outcome indicators cover a range of health conditions, including non-communicable, reproductive and childhood, and communicable diseases. With respect to UHC and health services, their analyses cover prevention (e.g. immunization) and treatments. The authors found different patterns between the two. One of their innovations with respect to the determinants was to introduce measures of interpersonal barriers to UHC that are close to the EQuAL Discrimination and Participation domains and the UHC concepts of people-centered care, for which data availability in the case studies was found to be relatively sparse. These ‘health systems responsiveness’ indicators were associated with poorer health and worse health service coverage, in particular for measles vaccination coverage in poorer populations. Another innovation was the use of an accountability policy variable, which was found to be statistically significant in several mortality regressions. The qualitative feedback from Blas et al. (27) and the literature review of Pedrana et al. (32) indicated that this was a less commonly studied area. If subnational variables on accountability could be identified, this analysis confirms that accountability indicators could be useful inclusions in national monitoring systems.

A paper by Pedrana et al. (32) reviews the literature from 2004 to 2014. The authors identified a final set of 96 articles of largely quantitative studies, most of which presented analyses of associations between a range of determinants with health and health coverage inequalities at the national level. The domains covered by the review studies tended to concentrate on socioeconomic indicators rather than domains related to processes (such as Accountability and Participation). The most frequent policy intervention themes that were addressed by the analyses of health determinants were health promotion and health literacy (25%), social protection (22%), and large socioeconomic development interventions (20%). There were also studies promoting anti-discrimination policies and occupational health services. New insights obtained from this review have implications for taking forward work on national monitoring standards. Many of the studies used explanatory models with multiple determinants as dimensions influencing health population outcomes. More nuanced definitions of individual and family socioeconomic status with measures and indexes of deprivation and financial hardship were noted. Other complex multidimensional measures of inequality considered inequity at different levels of social aggregation (individual level to family, neighborhood, community, municipal, and regional levels). Also, specific measures of inequality related to race, ethnicity, and gender were developed for the context of work or employment and family or social limitations.

Finally, a series of short communications describe on-the-ground national experiences of monitoring health determinants in England, Finland, and Mexico. In England, Goldblatt (33) stresses that monitoring has progressively shifted from a small set of targets to a wider set of determinants of the causes of ill-health and of health service performance. The paper emphasizes the experience of ‘localism’, referring to the need for monitoring systems to help find local solutions to local problems and to encourage community empowerment. Goldblatt argues that indicators need to be available at subnational levels and that methods need to rely on smaller in-depth surveys and cohort studies. This trend also implies the need to ensure harmony between local, national, and global monitoring efforts. In Finland, Kilpeläinen et al. (34) observe that monitoring covers a spectrum of information including with the purpose of evaluating the effects of health policies and interventions. In this regard, it is notable that the Finnish *Welfare Compass* provides indicators disaggregated at subnational levels on the provision of social and health services as well as the conditions (needs) of the households. The authors note that in spite of their good data, some health policy targets related to socioeconomic health differences have persisted. They conclude that data availability is not sufficient, but there is a need for wider dissemination and use of this information, in particular by political decision-makers and healthcare professionals. In Mexico, Martinez Valle (35) presents two case studies to show how public policies addressing health determinants with an equity focus have been monitored and evaluated, and how this has led to a better culture of monitoring and evaluation. The author describes how the monitoring of the *Prospera*, the living conditions of families in extreme poverty in terms of health, nutrition, education and income, has also helped policy-makers to improve the design and operation of this program. The monitoring of *Seguro Popular*, the financial health protection program, has helped to evaluate how financial barriers to health care have been reduced. The author says that evidence, legal mandates, and having a regulatory evaluation agency, have been fundamental to institutionalizing monitoring and evaluation in Mexico.

Taken together, this body of work provides evidence of the feasibility and validity of undertaking monitoring of the determinants of health. The papers highlight the importance of health determinants acting as barriers to UHC. The UHC barriers related to non-medical costs when accessing health services and completing health treatments, like transport and food, as well as inter-personal barriers associated with discrimination, communication, and other infrastructural or administrative requirements are important. These barriers to health service access were found to be inequitably experienced by disadvantaged populations in the case studies, and in the reviews of different countries (36). Although not being monitored systematically as yet, these types of barriers have received mention in the first global monitoring report for UHC (37).

Between 2015 and 2016, WHO has further advanced monitoring determinants through the development of a proposal for a global monitoring framework for action on social determinants of health (SDH) (38). This global monitoring framework is based on agreements made in the World Conference on Social Determinants of Health and is described in the Rio Political Declaration on Social Determinants of Health (39). Several measurement concepts and indicators proposed for global monitoring are common to the ones tested in national-level monitoring in the EQuAL framework (e.g. basic amenities coverage, health promotion expenditure, and social protection coverage). These areas emerged as strong priorities for action in the Rio Political Declaration. However, several other indicators of the Rio proposal differ from the EQuAL framework as the Rio Political Declaration focuses several measurement concepts on global level governance, and national-level policy interventions (40), rather than on the conditions of households (see Fig. 1). To make several of these governance indicators relevant for national policy-makers to assess within-country progress, new measurement approaches that assess subnational level implementation policies are needed.

To conclude, the range of domains, variables, and analytical approaches used and described in EQuAL demonstrates that promising opportunities exist for the development of national guidance on monitoring health determinants with an equity focus. Further work toward standards for national monitoring systems would need to take into account both the action (policies, programs and practices, including governance interventions) and the conditions experienced (26) by different populations.

In going forward, an interdisciplinary approach that learns from existing national monitoring frameworks and reviewing the evidence-base on indicator effectiveness, as well as from previous harmonization efforts in health promotion and environmental monitoring, will benefit the development of guidance for national and global monitoring. Building more effective national capacities to draw on and share data across different policy sectors and platforms, and to support within-country monitoring of health determinants with an equity lens, will promote achievement of the SDGs.

*Nicole B. Valentine* Department of Public Health, Environmental and Social Determinants of Health WHO, Geneva Email: valentinen@who.int*Theadora Swift Koller* Gender, Equity and Human Rights WHO, Geneva Email: kollert@who.int*Ahmad Reza Hosseinpoor* Department of Information, Evidence and Research WHO, Geneva Email: Hosseinpoora@who.int
